# Early mobility to improve health outcomes following cardiac surgery (EVE) clinical trial development: engagement methods using body mapping and virtual consensus building

**DOI:** 10.3389/fresc.2026.1801084

**Published:** 2026-10-08

**Authors:** Emily K. Phillips, Jacqueline L. Hay, April Gregora, Kathy Smith, Carly Shaski, Sarah Gilchrist, Andrew Fagan, Maureen C. Ashe, Annette S. H. Schultz, Rakesh C. Arora, Todd A. Duhamel, Sheila O’Keefe-McCarthy, Anna M. Chudyk

**Affiliations:** 1 Applied Health Sciences, Faculty of Graduate Studies, University of Manitoba, Winnipeg, MB, Canada; 2 Faculty of Kinesiology and Recreation Management, University of Manitoba, Winnipeg, MB, Canada; 3 Institute of Cardiovascular Sciences, St. Boniface Hospital Albrechtsen Research Centre, Winnipeg, MB, Canada; 4 University of Manitoba, Winnipeg, MB, Canada; 5 St. Boniface Hospital, Winnipeg, MB, Canada; 6 Max Rady College of Medicine, Rady Faculty of Health Sciences, University of Manitoba, Winnipeg, MB, Canada; 7 Department of Family Practice, The University of British Columbia, Vancouver, BC, Canada; 8 College of Nursing, Rady Faculty of Health Sciences, University of Manitoba, Winnipeg, MB, Canada; 9 Department of Surgery, Northwestern University, Feinberg School of Medicine, Chicago, IL, United States; 10 Department of Nursing, Faculty of Applied Health Sciences, Brock University, St. Catharines, ON, Canada; 11 College of Pharmacy, Rady Faculty of Health Sciences, University of Manitoba, Winnipeg, MB, Canada

**Keywords:** arts-based research method, body mapping, patient and pubic involvement, patient engagement in research, patient involvement and engagement

## Abstract

Patient engagement in research (PER) is increasingly recognized to enhance health research and facilitate the translation of research findings into practice. This occurs through integrating the knowledge, perspectives, and priorities of people with living/lived experience (PWLE) who receive or support those receiving care. Current approaches to PER emphasize verbal methods, where the thoughts and opinions of PWLE are shared in advisory groups, one-on-one or research group meetings, and town halls. While valuable, these methods often limit the expression of complex experiences, senses, and emotions that are difficult to articulate in words. Arts-based research methods involve individuals creating art, allowing for the exploration of embodied knowledge. Various types of art can be employed, including visual art, poetry, music, digital storytelling, and body mapping. Body mapping is an emerging arts-based research method that involves PWLE creating a life-sized tracing of their body in a position of their choice. The tracing is then filled based on reflections on guided questions about their healthcare experience using self-selected types of visual art and words. The knowledge gained from body mapping can be used to develop PWLE-informed recommendations on research directions, barriers and facilitators, and outcome and experience measures through subsequent virtual consultation workshops using the nominal group technique for consensus-building. While body mapping and virtual consensus building are promising approaches to generating future research questions and outcome measures, guidance related to their operationalization is lacking. This paper describes a model of a participatory approach to guiding research directions based on lived/living experiences through body mapping and subsequent virtual consultation workshops. The use of the model to explore the lived experience of early mobility after cardiac surgery while in the intensive care unit is used throughout as a case example. Using this model, we co-created an understanding of the embodied experience of early mobility after cardiac surgery in the intensive care unit, a patient-centred definition of early mobility, and generated and prioritized barriers and facilitators to early mobility along with outcome and experience measures to consider in future research. This model improves the accessibility of arts-based research methods for researchers engaging patients. In this way, researchers have more tools in their toolkit to broaden the types of knowledge that can be used to guide research and improve healthcare.

## 1 Introduction

Patient engagement in research (PER) is the meaningful and active engagement of people with lived experience (PWLE) of a health issue in research as members of the research team (1–3). In health research, PER is internationally recognized as important in generating knowledge to improve healthcare systems (1–3). PWLE can be engaged at any stage of research in grant development, intervention and outcome design, recruitment and data collection, analysis and interpretation (4) and contribute their unique lived/living expertise to the clinical knowledge of clinicians and the scientific expertise of researchers (1). Therein, their living/lived/living experience (herein referred to as lived experience) is integrated throughout the research process and used to inform and guide decisions, shifting health research to better align with the priorities of those receiving healthcare, which can also help overcome the lack of translation of research findings into clinical practice (1–3).

The dominant methods for PER, advisory groups, one-on-one or group meetings, and public or town halls, rely heavily on verbal communication (5, 6). Verbal approaches limit the knowledge available to that which can be verbalized, such as thoughts and opinions (7, 8), and exclude embodied knowledge, including senses, expressions, and emotions, that cannot adequately be put into words (6, 9, 10). Arts-based research methods have individuals create art to explore embodied knowledge (6, 9, 10). Removing the limitations of verbal expression allows for a richer sharing, understanding, and leveraging of the lived experience (6, 9, 10). Arts-based research methods are inherently participatory, offering PWLE the flexibility to choose how and what type of knowledge is shared (9).

Our research group recently published a narrative review exploring arts-based research methods used for PER, including how they can be used, at which research stages, at which engagement levels, and how they impacted the research (6). There are many arts-based research methods available for PER to access embodied knowledge, including visual art, poetry, music, digital storytelling, and body mapping (6). In particular, body mapping supports exploring the senses, emotions, and feelings of an experience (11–15). Body mapping can be used to engage PWLE to walk through a healthcare experience so they can relive it and access embodied knowledge to generate future research questions and outcome measures. PWLE create a life-sized tracing of their body in the position of their choosing (11–15). This body map is then filled in based on reflections to guided questions posed by the research team with paint, markers, crayons, collages, or words in whatever way is meaningful to PWLE to recreate their experience of a healthcare event (11–15).

A goal of PER is to align research directions with the priorities of PWLE (1–3). One way of doing this is to generate and prioritize research directions through virtual consensus-building workshops (16, 17). Existing models for understanding and integrating the priorities of PWLE in future research directions have focused on didactic engagement methods (16, 17). These generate concepts which are then analyzed, organized, and presented to PWLE, who then prioritize them (16, 17). Body mapping generates embodied experiences that share knowledge that is difficult to verbalize (11–15). Thus far, researchers have presented these experiences as research findings but have not described or demonstrated how to use them in future research (6). This limits the utility of body mapping for PER, as the ability to use this research method to meet a goal of PER has not been demonstrated.

Although the current PER literature supports the integration of body mapping conceptually (6), models for operationalizing it are lacking, which is a barrier to its use. There is also a lack of guidance on how to transform embodied knowledge into tangible future research directions. Establishing models would increase the accessibility of body mapping for PER, in addition to improving the accessibility of PER for arts-based researchers to enable the generation and use of multiple sources of embodied knowledge to produce research aligned with lived experiences (6). Previous investigations have largely been limited to the utilization of body mapping for data collection and some analysis only (11–15). We extend this existing knowledge base by providing a participatory approach to guiding research directions based on lived experiences through body mapping and virtual consensus workshops. The primary objectives of this methods paper are to provide a model for operationalizing body mapping as a participatory research approach and to share how the knowledge gained from body mapping can be used to generate future guiding research directions.

This article describes a case study that can serve as a model for operationalizing the integration of arts-based practices to engage PWLE in research, detailing how researchers and PWLE can collaborate to create body maps, analyze them together, and co-synthesize the data produced to generate future research questions and outcome measures. Thus, it presents an arts-based participatory approach to data collection, analysis, synthesis, and prioritization driven by the lived experience. We used this model to successfully explore embodied knowledge of the lived experience of early mobility after cardiac surgery while in the intensive care unit (See Box 1). This is the first article in a three-part series that describes a two-phased workshop to develop a PWLE-informed early mobility intervention for use in the cardiac surgery intensive care unit (CS ICU). The following two articles describe the findings of a study that employed this model to co-create an understanding of the embodied experience of mobility in the CS ICU, co-create a definition of early mobility, identify barriers and facilitators to early mobility, and prioritize outcome and experience measures for use in future intervention research. This work is part of the Early mobility to improVE health outcomes following cardiac surgery (EVE) Clinical Feasibility Trial Development study.

BOX 1 **Information about the case example.**

**Table:** 

Post-intensive care syndrome (PICS) is a new or further deterioration in cognitive, mental, and/or physical health after admission to the intensive care unit (ICU) (18). Between 16% to 99% of patients demonstrate symptoms of PICS in the cardiac surgery (CS) ICU (19). Interventions aimed at preventing PICS are required to support optimal health outcomes for these patients. Early mobility interventions in the CS ICU improve physical health outcomes (20–22), are safe (23–28), and are a guideline recommendation (29, 30). Despite an increasing research focus on early mobility in the last decade (31), there remains a lack of integration of early mobility into CS ICU clinical care (32, 33). Research teams investigating early mobility interventions thus far have been dominated by physicians, nurses, and physiotherapists, with rehabilitation scientists and exercise physiologists less frequently involved (31). None of the research teams have included PWLE of CS. A team approach is required, with collaboration among clinicians, PWLE, and researchers, to develop early mobility interventions and support selecting outcomes relevant to PWLE. The research this model was used for begins to address these knowledge gaps to improve CS ICU patient care by exploring the embodied lived experience of early mobility in the CS ICU. The objectives of the study underlying this model were: (1) co-create an understanding of the experience of mobility in the CS ICU, (2) co-create a definition of early mobility, (3) identify barriers and facilitators to early mobility, and (4) select outcome and experience measures for use in future early mobility intervention research.

## 2 Methods

The described model involves a sequential series of co-facilitated workshops to co-create, co-analyze, and co-synthesize body maps and discuss and prioritize experiences shared during the co-analysis to generate future research questions and outcome measures. Reporting of patient engagement in this work follows the updated Guidelines for Reporting Involvement of Patients and the Public (GRIPP-2) long form (see supplemental material) (34). This work was conducted in alignment with the Tri-Council Policy Statement: Ethical Conduct for Research Involving Humans—TCPS 2 (35). Institutional review board approvals for the case study exemplar were obtained in March 2024 from the University of Manitoba Research Ethics Board 1 (HE2024-0035) and the St Boniface Hospital Research Review Committee (RRC/2024/2158). All PWLE reviewed and signed a consent form prior to participating in the workshops of the described case study. PWLE were made aware that consent was an ongoing process, and they could withdraw consent until data analysis began, at which point it was no longer possible to remove their data from the study. No PWLE withdrew their consent.

### 2.1 Patient engagement in this paper

The approach to engaging PWLE is informed by the Canadian Institutes of Health Research's Strategy for Patient-Oriented Research Framework (36) and our research teams' previous work (7, 8, 16, 37). The PWLE research team members, AG and KS, collaborated (38) through the entire project and were involved in conceptualization, development and conducting the case study exemplar, which originated our model. Additionally, AG and KS supported drafting the initial version of this paper and helped revise subsequent versions. As noted in previous work by members of the research team, the engagement of AG and KS in the conduct of the case study exemplar “helped to bridge the divide” between clinician and academic research team members and the workshop PWLE (16).

### 2.2 Overview of the model

The model's overarching purpose is to guide research directions and outcome measures through the co-creation, co-analysis, and co-synthesis of body maps pertaining to the healthcare experience under study. The first step involves identifying the research direction(s) (and underlying research question[s]) to be addressed by the process. Workshop 1 (co-creation) involves three in-person sessions in which PWLE create and discuss body maps of the healthcare experience under study. The research team then collates discussions to support co-analysis and the determination of outcome measures. Workshop 2 (co-analysis and co-synthesis) involves one virtual session in which PWLE discuss and develop PWLE-informed recommendations on the research directions under study, potentially including related barriers and facilitators, along with outcome and experience measures. Workshop 3 (co-synthesis and prioritization) involves one virtual session where the recommendations (and/or their underlying facets such as barriers and facilitators) and the outcome and experience measures are prioritized using the nominal group technique (8, 39–43). Two rounds of prioritization occur, with a discussion between rounds focused on the initial prioritization from least agreement to most agreement (8, 39–43). Workshops 2 and 3 are held virtually to reduce the burden of travel for PWLE. Figure 1 shows a visual overview of the model. Table 1 provides an overview of the goals and outputs of the workshops that comprise the model. Each workshop built on the prior, generating and interpreting knowledge simultaneously and collaboratively.

**Figure 1:**
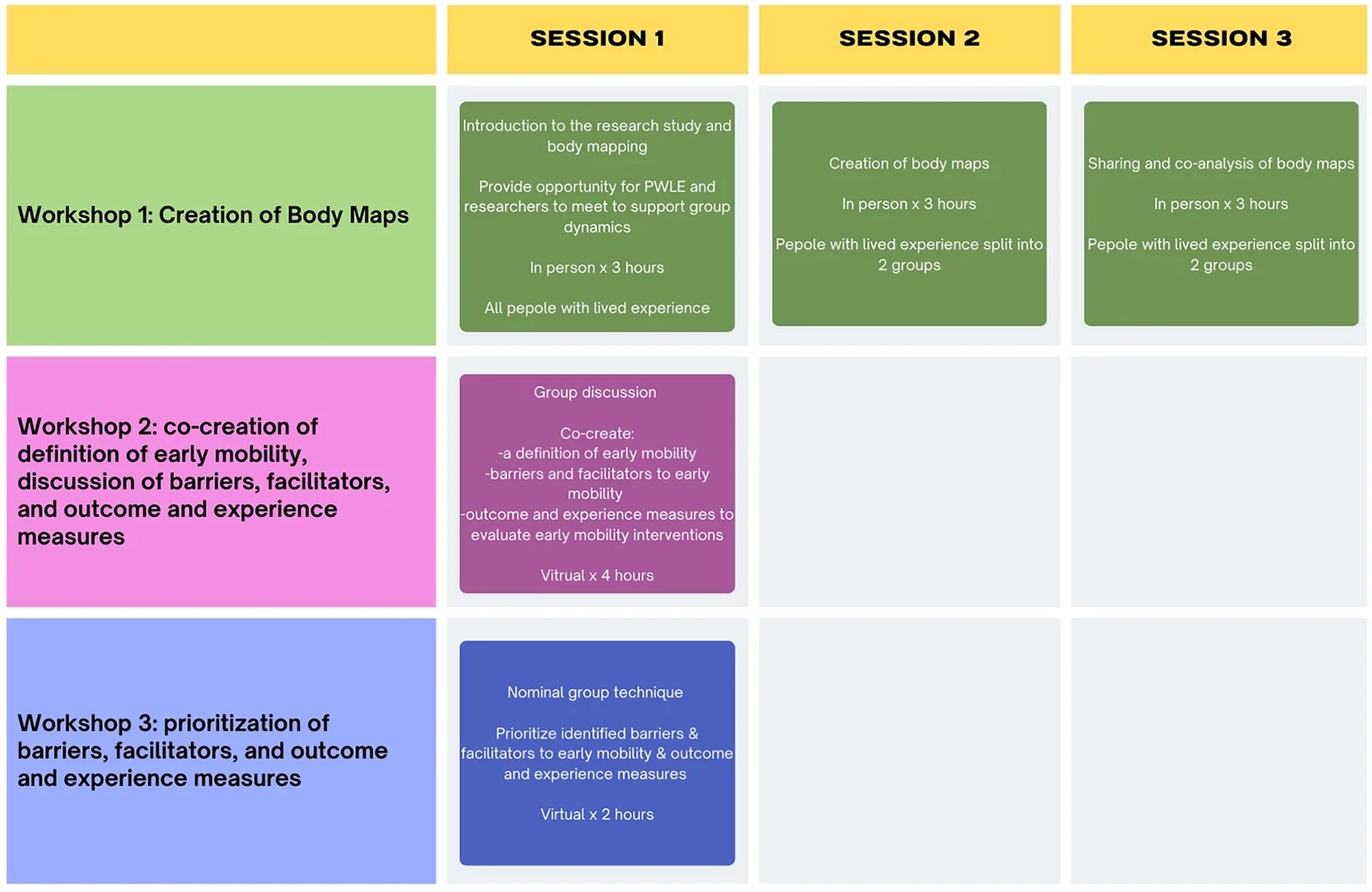
Visual overview of the workshops and sessions.

**Table 1:** Goal and desired outputs of each workshop.

Workshop number		Goal	Desired outputs
Workshop 1: Creation and Description			
1	Session 1	Develop group dynamics Introduce research questions Introduce body mapping	Developing team cohesion Understanding of research project Understanding of body mapping
	Session 2	Create body maps	Body maps
	Session 3	Present body maps	Initial analysis of body maps
Workshop 2: Co-analysis and Synthesis			
2		Co-create a definition of early mobility Identify barriers & facilitators to early mobility, and outcome & experience measures	Definition of early mobility List of barriers & facilitators to early mobility and outcome & experience measures
Workshop 3: Co-synthesis/Prioritization and Recommendations			
3		Prioritize barriers & facilitators to early mobility, and outcome & experience measures	Prioritize list of barriers & facilitators to early mobility, and outcome & experience measures

### 2.3 PWLE recruitment

The suggested sample size is 8–12 participants, which aligns with recommendations for optimal group size to facilitate discussion and participation (44). Recruitment can occur through multiple avenues, such as patient and family advisory councils, databases of patients who have consented to be contacted for future research, or through networks of PWLE research team members. Inclusion and exclusion criteria should be developed that reflect the lived experience of interest. For example, limiting recruitment to people who have undergone certain procedure types may be important if these procedure types lead to differences in the living/lived experience of interest. Equity, diversity, and inclusion (EDI) considerations should also reflect the living/lived experience of interest. Exploring the living/lived experience based on different EDI factors in separate studies may be beneficial in guiding research directions. Thoughtful reflection should be used when considering inclusion and exclusion criteria and EDI.

Each participant should be compensated for their time. The Strategy for Patient-Oriented Research Recommendations on Patient Engagement Compensation can be used to guide compensation (45). These recommendations suggest $25 per hour for engagement up to four hours at a time (45). The time commitment should include the time required to complete all workshop sessions and preparatory work. Research teams should check with their local ethics review board as to whether ethics approval is required for workshop participation, especially if they intend to publish on the process and/or its findings. See Box 2 for an example of recruitment used for the case exemplar.

BOX 2 **Recruitment in the case example.**

**Table:** 

We recruited 10 participants in alignment with recommendations for group size to facilitate discussion and participation (44). Participants included patients and their care partners (family or friends). The inclusion of care partners was suggested by AG and KS, who shared the difficulty of remembering the ICU hospitalization and the key role care partners play in helping to piece together memories of the time. Patients and care partners were recruited from a database of patients who have undergone CS at St Boniface Hospital in Winnipeg, MB and who agreed to be contacted for future research. Inclusion criteria were adults over 18 who have undergone or had a loved one undergo elective or semi-urgent outpatient coronary artery bypass grafting, valve CS, or a combination of the two at St Boniface Hospital in the last five years, cognitively able to participate in a consultation workshop, had access to the internet and an electronic device, were willing to reflect on personal experiences relating to CS and share these in a group setting, could speak and read English, and consent to be recorded. Each participant was compensated $475 for their time. This amount was based on the Strategy for Patient-Oriented Research Recommendations on Patient Engagement Compensation (45). These recommendations suggest $25 per hour for engagement up to four hours at a time (45). We estimated that 19 h would be required to complete all workshop sessions (which total 15.5 h) and preparatory work. All participants provided written informed consent prior to workshop participation. All in-person sessions were held at St Boniface Hospital in the same building that houses the CS ICU. Locating the study as such was intentional to elicit thoughts and emotions through the familiar environment.

### 2.4 Detailed model description

#### 2.4.1 Preparation

A standard operating procedure (SOP) should be created ahead of the workshops to guide each session (see Table 2). The SOP can be pretested by having research team members create their body maps. These are valuable examples for PWLE to understand the request. This also allows inexperienced facilitators to gain more familiarity with body mapping. Finally, this allows the standard operating procedure to be piloted to ensure the instructions are clear and each facilitator has a clear understanding of their roles (see Supplementary Materials). See Box 3 for an overview of the model from the case exemplar.

**Table 2:** Items to include in the standard operating procedure.

Item	Description
Overall goal of the workshops	– The overarching goal of research, likely specifying the future research questions and outcome measures of interest
Goals and outputs of each workshop and its sessions	– The sub-goals of each session that together culminate in the overall goal – Outputs can be tangible, such as a body map, or co-developed knowledge, such as a definition of early mobility
List of materials that will be provided to participants before each workshop (Materials should be provided to participants at least one week in advance of a session to support adequate time to reflect and determine which elements of their experience they would like to share and how they would like to share them)	– Any documents that will be shared, such as a map to the workshop location, a list of suggested attire, and questions that will be posed at the workshops for the participants to consider – A questionnaire should be given to participants to complete ahead of time to identify any accessibility and other needs – Minimal information about body mapping can be provided prior to the workshop sessions with the assurance that examples will be provided and there is no correct way to create a body map
Detailed plan for each workshop session	– From welcomes and introductions to the activities of each session – May outline the time each section will take and which facilitator is responsible to lead each section

BOX 3 **Overview of the model actioned in the case example.**

**Table:** 

Workshop 1 The words generated by PWLE of their physical, mental, and emotional experiences and feelings related to early mobility in the CS ICU were recorded and posted in the workshop room throughout the 3 session days to allow PWLE and facilitators to reflect on them. Probing questions used to explore the body maps included asking why specific colours were used (for example, why red was used for pain or why bright colours were included) and why specific images were used (for example, why dumbbells were included or why rabbits were included). Preparation for Workshop 2 This is the summary shared with PWLE prior to Workshop 2: Summary of Body Mapping June 4–6, 2024
Experience of early mobility Foggy/ fuzzy memory Start with a body scan—am I alive, am I in pain, am I ok? Being able to move means survived surgery! Lots of tubes and IVs and things to manage Lots of pain, mostly not from the surgery (not chest or leg) Frustration with not being independent Knew would be tough to do but necessary part of recovery Want to get home so this is an important early step Need encouragement and help to mobilize (related to pain, lack of motivation, all the lines and tubes) Keeping a positive attitude helps Easy to feel embarrassed or awkward
Early definition Day of surgery 5–6 h after surgery Day after surgery 2 days after surgery Not until out of ICU Person specific! Tie to assessments and meeting milestones rather than a specific timeframe
Mobility definition Sit and stand at the side of the bed Sit in a chair/ commode Move arms and legs in bed Walk
Barriers to early mobility Brain “fuzziness” Weakness Fear Unclear source of pain “Paraphernalia” Poor sleep/ food/ appetite Lack of energy
Facilitators of early mobility Positive attitude Being encouraged/ forced/ helped IV pole Messaging that mobility means getting out of “this world” (hospital) and back to your world Education about comorbidities interacting and causing unclear discomfort Seeing others moving and leaving hospital Education about post-operative care Being given a choice of when to mobilize Being given repeated reminders to mobilize Good food Confidence in team and system due to team explanation and history of success of program Workshop 2 In Workshop 2, PWLE discussed the definitions of early, ranging from the day of surgery to not until out of ICU. PWLE came to the consensus that the definition of early needs to be person-specific and tied to patient assessments rather than a specific post-operative timeframe. PWLE then discussed the definition of mobility, which ranged from sitting and standing at the side of the bed to ambulation. PWLE came to the consensus that mobility needs to be defined on a spectrum of activities that reflect how they need to move to go home. They then generated barriers, facilitators, and outcome and experience measures, as shown below. Outcome measures were grouped into cognitive, mental, and physical outcomes to align with the components of PICS.
Barriers Brain “fuzziness” Weakness Fear Unclear source of pain “Paraphernalia” Poor sleep/ food/ appetite Felt not like self, uncertain of what capable of, not weak necessarily, not sure if can do what being asked to do
Facilitators Positive attitude Being encouraged/ forced/ helped IV pole Messaging that mobility means getting out of “this world” (hospital) and back to your world Education about comorbidities interacting and causing unclear discomfort Seeing others moving and leaving hospital Education about post-operative care Being given a choice of when to mobilize Being given repeated reminders to mobilize Good food Confidence in team and system due to team explanation and history of success of program No pain (minimally invasive cardiac surgery) Staff—kindness, supportive Engaging with family and friends
Outcome Measures Physical Light-headedness/ dizzy SOB Confidence in moving (related to packing and incisions) re bleeding or bumping it No remember doing ADLs in ICU (they did it all), on the ward they brought stuff to the chair to do there, felt good to go do it “for real” in the bathroom Cognitive Do you feel foggy? Do you feel confused? Are you seeing anything strange/ odd/ hallucinating? Care partner question for sure—do they seem confused/ are they hallucinating Mental No symptoms Are you focused on the positive or the negative?
Experience Measures Care and attention from nurses and doctors MD coming into see even when not working Making feel like you matter Nurses being attentive and helpful not making feel like a burden Not being made to feel like a bother when ringing for help Preparation for Workshop 3 The barriers, facilitators, and outcome and experience measures were used to create a survey in Microsoft Forms by EKP. This survey was sent to JLH, AG, and KS to pilot test and ensure instructions for completion were clear. Workshop 3 The group discussion led to several wording changes in the survey, which improved the agreement when the second survey was shared.

### 2.5 In-person workshop overview and key considerations

#### 2.5.1 Workshop 1

Workshop 1 consists of three in-person sessions. The in-person format is important for creating and sharing body maps as it allows facilitators to support each PWLE appropriately. It also allows the research team to provide all art supplies feasibly, rather than sharing these through home delivery or mail. It is recommended that the group of PWLE be broken into two groups, such as a morning and an afternoon group, for Sessions 2 and 3. This ensures facilitators can provide appropriate support to each PWLE while creating and discussing their body maps. Each of the sessions is approximately four hours long. This provides adequate time to build relationships and trust and learn about body mapping (Session 1), create a body map (Session 2) and provides each PWLE approximately 30 min (assuming 6 PWLEs in each group) to present their body map and for the group to take a break (Session 3).

##### 2.5.1.1 Session 1—relationship building

The goals of Workshop 1 Session 1 are to build relationships among PWLE and research team members, to introduce the arts-based research method of body mapping, and to support PWLE in exploring their experiences, emotions, and feelings related to the healthcare experience under study. The session begins with introductions and an icebreaker activity, preferably aimed at sharing meaning and experiences and highlighting how each person brings a unique perspective to the same question. For example, an icebreaker activity could involve having every attendee share a small item that is important to them and explain its significance. In the next activity, the group takes turns sharing words to describe their physical, mental, and emotional experiences and feelings related to the healthcare experience under study. A session facilitator writes these in coloured marker on a large sheet of paper posted in the room. This activity is meant to help PWLE create their body maps by reminding them of the feelings they may want to depict on their body map. The example body maps created by the research team are then shown to PWLE. These provide a tangible example of what PWLE will be creating. Sharing multiple body maps is beneficial as it demonstrates that there is no “right way” to create a body map. Instead of creating body maps, the research team could share examples from the literature. Finally, PWLE are shown the art supplies available for creating their body maps. They are then asked to think about the position they want their body in on their map, what colours they want to use to depict their experiences, and what images they want to use to depict their experiences (for example, how will they depict feeling unsteady on their feet or feeling confused). A key consideration for Session 1 is ensuring enough time to support team building through the introductions. Based on our experience with the case exemplar, we recommend 5 min per person, which may seem like a lot but helps support the establishment of a cohesive group that works well together and supports one another over the following workshops and sessions.

##### 2.5.1.2 Session 2—creating body maps

The goal of Workshop 1 Session 2 is to create body maps. Facilitators (one per PWLE) support PWLE in tracing an outline of their bodies and help them reflect on their experiences, emotions, and feelings about the research direction/question being addressed. This supports PWLE to fill in the body maps to depict. Facilitators support reflection by talking to them about their relevant experiences and feelings and how to potentially depict them. They also work with PWLE to create a title for their body map. A key consideration for Session 2 is to provide PWLE the space to request as much or as little facilitator support as possible. Some PWLE will come with a vision of their body map and may want very little support. Others may be unsure of how to start creating their body map, or how to fill in specific details, and need encouragement and understanding that there is no wrong way to express ideas.

##### 2.5.1.3 Session 3—begin co-analysis

In Workshop 1 Session 3, the goals are for each PWLE to share their body map with the group and to begin to co-analyze it using compositional interpretation (46). The body maps should all be displayed on one wall, with one brought forward for sharing at a time. This allows PWLE to listen to the questions being posed and reflect on their body map to support their discussion. The body map being shared is taped on the wall between the PWLE and one facilitator who leads the discussions. To introduce the PWLE to what the discussion could look like, at least one PWLE research team member should discuss their body map first to decrease uncertainty/increase comfort about the process. The PWLE team member could create this body map outside of the workshop or during Workshop 1 Session 1. PWLE are then asked to volunteer to discuss and co-analyze their body maps using compositional interpretation (46). With this analysis approach, the content, colour, spatial organization, light, and expressive content of the body map are co-analyzed through a facilitated discussion (46). The analysis of the content of the image includes discussing what is shown and what symbols or icons are used (46). When analyzing colour, the dominant hue, colour saturation, the lightness or darkness of colours, the harmony of the colours used, and what is stressed with colour are investigated/discussed (46). Spatial organization looks within the image at the direction of lines. If the image is static or dynamic and beyond the image to include the point of view the image is created from (46), while the analysis of light explores the use of light to highlight elements of the image (46). Finally, the co-analysis of the expressive content of the image is the emotive effect of the image (46). These elements are what the facilitator focuses on discussing to ensure the correct interpretation of their meaning for PWLE. This co-analysis ensures the meaning intended by PWLE is captured, rather than the research team's interpretation of the body map.

PWLEs' presentations of their body maps and ensuing conversations can be video recorded along with a video recording of the other PWLE watching the discussions. The video recordings can be used to support preparation for Workshop 2 and for future secondary data analysis. Video recordings allow for the consideration of body language, facial expressions, and gestures at specific items on the body map that would be missed if using audio recordings alone.

A key consideration is to allow the discussion to follow the direction of the PWLE. Each experience is unique, so different prompts and questions are required to truly explore each. Potential probing questions to explore the body maps include asking about why specific colours and images were used. This exploration of the thought and meaning attached by each PWLE to their body map is the beginning of participatory data analysis as participants are engaged in exploring the meaning of the data they created (i.e., their body map).

### 2.6 Virtual workshops

#### 2.6.1 Preparation for workshop 2

In preparation for Workshop 2, the research team summarizes the experiences shared during the co-analysis of the body maps (from Workshop 1, Session 3). It provides recommendations on the research directions under study, including barriers and facilitators identified by PWLE. The recordings of the discussions of the body maps can be used to guide the creation of the summaries as required. Summaries should rely on the words PWLE used and not an interpretation of them. For example, if a PWLE described “seeing things,” this phrase should be used and not changed to “hallucinations.” The summaries can simply list the experiences shared; these do not need to be grouped by themes or analyzed in any way. The goal of the summaries is to expose all PWLE to the experiences of members of the group they were not part of due to the group's division. As well, an overview of any relevant research directions or outcome measures that may not be familiar to PWLE should be shared. For example, an overview of outcome and experience measures explaining each and providing examples can support PWLE in understanding the workshop content and allow them to reflect and prepare for the workshop.

#### 2.6.2 Workshop 2 goal and overview/key considerations

Workshop 2 is held approximately one week after Workshop 1 to allow for the generation of the summaries described above. The workshop is approximately four hours long. Further co-analysis and co-synthesis of the experiences depicted in the body maps are the goals of the workshop. These goals are met through facilitated discussions, each focused on a subset of the research directions (and/or their underlying barriers and facilitators) and on outcome measures.

The experiences depicted in the body maps are discussed to more deeply co-analyze and co-synthesize a unified experience as guided by the PWLE. Co-analysis begins using the summary of the research directions to guide the discussion. Experiences are discussed to explore their meaning, similarities, and differences. Co-synthesis occurs through the crafting of a narrative that weaves the shared experiences together into a cohesive story. PWLE then generate outcome and experience measures related to the guiding research directions through a discussion where PWLE share what outcome and experience measures are important in evaluating the research direction of interest. Co-synthesis again occurs when the research team and PWLE group common outcome and experience measures. Specific tools are not necessarily discussed, as the focus is on understanding the meaning of the outcomes and experiences discussed. If part of the research direction, barriers and facilitators described in the body maps are discussed, and PWLE can make additions. Co-analysis begins by exploring the meaning of the barriers and facilitators to PWLE. This supports co-synthesis by supporting the research team and PWLE in grouping common barriers and facilitators together.

There is one key consideration for Workshop 2: technological support. One facilitator should be designated for this role. They will collaborate with PWLE to ensure they can fully access the virtual platform, including the ability to mute and unmute their microphone.

#### 2.6.3 Preparation for workshop 3

Following Workshop 2, a survey listing the research directions (including barriers and facilitators if relevant), as well as the outcome and experience measures, is created. This survey will be used in Workshop 3 for PWLE to prioritize the recommendations. It can be pilot-tested with the facilitators to ensure it functions as intended by the team.

#### 2.6.4 Workshop 3—prioritize research direction

Workshop 3 occurs approximately 2 weeks following the previous workshop to allow for reflection, survey creation, and testing. It is approximately 2.5 h long to support time for prioritization and discussion. The goal of Workshop 3 is to prioritize the research directions (including barriers and facilitators if relevant) and the outcome and experience measures using the nominal group technique (8, 39–43). At the start of the virtual workshop, the prioritization survey is shared with the PWLE through email. The PWLE are instructed to complete the survey independently in a given time period (typically less than 30 min to facilitate discussion and subsequent re-prioritization). All responses are anonymous. After all PWLE complete the survey or the allotted time to complete the survey has run out, the responses are reviewed by the research team to support the upcoming group discussion. Using survey software that analyzes and ranks the responses, such as Microsoft Forms, is ideal. The research team's review focuses on the ranking of responses and the identification of the most agreed and least agreed upon responses (8, 39–43). For example, if a research direction is ranked highly by all PWLE, it would be widely accepted. If some PWLE ranked a research direction high and others ranked it low, it would be less agreed upon.

When all PWLE have rejoined, a discussion occurs that begins with the least agreed-upon research directions. PWLE discuss how and why they prioritized these research directions in relation to others. This discussion may lead to rephrasing research directions for clarity or consistency with the lived experience and combining similar research directions. The discussion moves to the most agreed-upon research directions, which usually require minimal editing. This process occurs again for the outcome and experience measures, as well as barriers and facilitators if relevant.

As these discussions occur, a research team member simultaneously creates a second survey. This survey captures all the changes (e.g., rephrased or combined research directions) from the discussions of the first survey results. After the first survey discussion is complete, or there are approximately 30 min left in the workshop, the second survey is shared with PWLE to complete, following instructions similar to the first survey. Time permitting, the findings of the second survey are shared with the PWLE, after which the workshop ends.

There are two key considerations for Workshop 3. The first is technological support. This role was again assigned to one facilitator, and they also supported the creation and revision of the second survey. The second is using a survey platform, such as Microsoft Forms, that automatically analyzes responses. This allowed the facilitators to review the prioritizations and quickly identify where responses aligned and diverged. If such software is not used, a longer time will be required for this Workshop to allow facilitators to analyze the responses for agreement.

### 2.7 Post-workshops

Following the completion of all workshops, the prioritized recommendations for research directions can be used to inform future research. See Box 4 for an example of how experiences can inform future research. The prioritized outcome and experience measures can also be used to inform the evaluation of future research. The outcome and experience measures identified provide a roadmap for the evaluation components that are important to PWLE. These could be incorporated as primary and secondary outcome measures in future research. If relevant, barriers and facilitators can be used to identify areas of focus to support the implementation of future interventions. Finally, narrative inquiry can be conducted using video recordings of the body map facilitated discussion. This deeper analysis can compensate for the shortcomings of compositional interpretation, specifically the lack of consideration for the production of the image and its interpretation by a broader audience (46). A narrative inquiry approach allows for data and investigator triangulation and the inclusion of interpretation by multiple research team members, along with the image creator. This analysis can include PWLE to support the findings, retaining the lens of embodied knowledge of their lived/living experience. The findings from a narrative inquiry should be used alongside the co-analyzed and co-synthesized findings to drive/inform future research directions.

BOX 4 **Post-workshops.**

**Table:** 

In the case example, the experiences are being incorporated into the development of an early mobility intervention. For example, the extent of brain “fuzziness” and the experience of early mobility meaning survival and moving towards getting home is being incorporated into education about supporting early mobility in the CS ICU. The top three barriers and facilitators are also being incorporated into the developing early mobility intervention. The outcome and experience measures identified will be used to guide the development of the evaluation of this early mobility intervention. The shortcomings of compositional interpretation were balanced through narrative inquiry (9) of the transcripts of the workshop discussions and participant observation (47) of the videos of the body map presentations. Narrative inquiry focuses analysis on plotlines, thematic structures, and social and cultural references, rather than on the smaller scope of words and phrases as in other qualitative methodologies (48). It allows for richer descriptions of shared experiences (9). Narrative inquiry follows the typical thematic analysis process of developing codes, identifying patterns among codes, and organizing the codes into categories and themes (48). Narrative inquiry was conducted by three academic research team members and the two PWLE research team members. To ensure that PWLE's gestures and body language were captured, the videos of PWLE presenting their body maps and of the other PWLE watching the presentations were used as sources of data. Participant observation aimed to incorporate tone of voice and facial expression with the body maps and discussions with PWLE. This non-verbal communication accounts for over 90% of the communication of emotions (49), so using participant observation of the videos supported a richer analysis. Participant observation was conducted by two independent researchers to mitigate researcher bias. To align with ethical requirements, videos were stored on the university-approved platform that is password protected. They were saved with coded file names and access to the videos was limited to those research team members who facilitated the workshops to support anonymity.

## 3 Discussion

In this article, a participatory model for patient and care partner engagement was introduced that extends Oravec et al.'s (16) model (integrating patient perspectives in research) with the addition of arts-based practices to maximize polyvocality to ensure that research-informed interventions are directly relevant to those with lived experiences. We assert that using arts-based participatory approaches to design research questions, conduct data collection or perform analysis and synthesis assists in operationalizing the collective voice of all team members effectively. In our model, data were collected and analyzed through the creation and discussion of body maps that depict the experience, emotions, and feelings related to a research question. The initial analysis was then synthesized through virtual workshops to generate tangible and novel contributions to the extant body of literature and current practice(s). This model is novel, as there is a lack of guidance for using arts-based research methods beyond data collection for PER (6).

This model builds upon an existing model for integrating patient experiences into research analysis and interpretation (16). It describes a process to generate and prioritize the embodied experience of a research question (16). The research question of the case example of the model described in this article focuses on embodied knowledge concerning senses, experience, emotions, and feelings, which are less easily put into words (6, 9, 10, 50). Different research methods exist to explore different research questions (51, 52). The research method should align with the research question (51, 52). Therefore, a variety of research methods must exist and be accessible to researchers. The current model offers access to a research method that can be used to explore research questions centred on the embodied knowledge of lived experience, senses, emotions, and feelings rather than thoughts and opinions.

Integrating lived experience into CS research is valuable and has been demonstrated in several studies. One example is the development of a mobile health application to support enhanced recovery protocols (53). Engaging PWLE led to key design and content elements that would have been missed without their engagement (53). Tailoring the design and content to the needs of PWLE is logical, as the end users of the mobile health application. Similarly, there is logic in engaging people who have undergone CS to determine care preferences and outcomes. PWLE have preferences and prioritized outcomes that both align with and diverge from clinicians and researchers (7). For example, a scoping review and virtual consultation workshop identified 140 patient and caregiver preferences and 19 outcomes (7). The workshop participants generated 21% of the patient and 66% of the caregiver preferences included in the study results (7). Said another way, one-fifth to two-thirds of lived experience preferences were missing from the literature prior to this publication. Similarly, 22% of the patient outcomes and 100% of the caregiver outcomes were generated from the workshop (7). Including PWLE adds value to cardiac surgery research by ensuring that research aligns with the priorities of PWLE, the end users of cardiac surgery. Finally, there are 22 enhanced recovery recommendations following cardiac surgery (8, 29). Implementing so many recommendations simultaneously is challenging for many cardiac surgery centers, particularly when they are implemented all at once (8). Using the nominal group technique, PWLE identified the priority recommendations for pre-operative, intra-operative, and post-operative time points (8). This supports aligning clinical care with the priorities of the end users of healthcare, PWLE (8). Expanding engagement from perspectives, thoughts, and opinions to include senses, experiences, emotions, and feelings will further align cardiac surgery research with the end users of cardiac surgery and further improve the care they receive. The model described in this article provides such a participatory methodology.

### 3.1 Key considerations

#### 3.1.1 Time

The workshops in the model described in this article were completed over a 4-week period. This amount of time was required to allow for the collation of the initial analysis from Workshop 1 and to support institutional ethical requirements. When planning for PER, realistic timelines are required as meaningful engagement does extend the time to complete research (54). This time investment is well spent when the research results better align with the end users of the research, PWLE.

#### 3.1.2 Money

The model described in this article includes compensation of PWLE for their time. This requires funding. Suggestions for compensation and differentiating compensation from reimbursement exist (45). These should be reviewed before submitting a grant to ensure the budget accurately reflects the compensation suggested (55). It is also important to discuss compensation options with PWLE. Compensation can impact income level and may lead to issues with social support services (56). Early and open conversations about the amount and form of compensation are required to ensure that compensation is not a barrier to engagement.

#### 3.1.3 Safeguards to reliving an experience

Exploring experiences, emotions, and feelings through arts-based methods can cause participants emotional stress (6). Appropriate supports are required for PWLE who struggle with reliving their experiences, emotions, and feelings (6). These supports can range from the facilitators, who can move the PWLE to a physical or virtual breakout room and speak to them to determine next steps, to accessing external supports such as family, a healthcare provider, or resources from local mental health services. If a PWLE is in distress, they should be directed to their nearest health facility. Researchers can consider including a psychologist and referral protocols available if deemed necessary. Art therapy has been used to support people in healing from traumatizing experiences, so the process of using art for engagement may be both stressful and cathartic (6). Regardless, open communication among facilitators and PWLE is required to support PWLE while reliving their experiences, emotions, and feelings.

### 3.2 Limitations

The model has several limitations. First, it was developed based on a single-center study. Further research is needed to explore the adaptability to other contexts. This model is likely transferable beyond the CS population. Body mapping has been used in community and outpatient settings (12, 13, 15, 57, 58) and as a research methodology with people with differing intellectual abilities (59). This suggests that Workshop 1 is accessible for many contexts. Second, the model may exclude PWLE who do not feel that are able to create art. This was noted when recruiting PWLE for the case example. It was overcome with some PWLE through conversation about body mapping and the process that would be used. Third, the model may exclude PWLE who lack digital literacy. Support may be required to facilitate participation in the virtual workshops for some PWLE and in some contexts. For example, virtual platforms may not be accessible for some PWLE due to socioeconomic factors or low digital literacy (16, 17). There has been a growth in the use of virtual platforms since the COVID-19 pandemic (16, 17), suggesting that a context-specific needs assessment is warranted to adapt the model to suit different populations. Finally, facilitators with experience in discussing and managing emotions and feelings and in engaging PWLE are required. This model uncovers deep emotional knowledge, and facilitators must be ready to engage in conversation about this and support PWLE as necessary.

## 4 Conclusion

Ensuring maximal buy-in from the ultimate end user of research-informed interventions impacting/affecting patients' and care partners’ lives requires creative methods to engage PWLE in research. Engaging PWLE is integral to generating research evidence that aligns with patient and care partner priorities and generating knowledge to improve healthcare systems (1–3). Verbal methods for PER are valuable to learn thoughts and opinions (7, 8). Exploring the layered complexities of embodied knowledge (senses, experiences, emotions, and feelings) of a health-related issue requires different methods, arts-based methods, such as body mapping (6, 9, 10, 50). Using novel methods required an understanding of the process. This article describes the process of using an arts-based participatory methodology for data collection, analysis, and synthesis. Co-creating body maps, co-analysis, and co-synthesis of the data leads to tangible and novel contributions to the literature that focus on embodied knowledge of the senses, experiences, emotions, and feelings of PWLE. These can be transformed into future research directions to align health research with the goals of PWLE (1–3).

## Data Availability

The raw data supporting the conclusions of this article will be made available by the authors, without undue reservation.
