# Supplementary material for: Early mobility to improve health outcomes following cardiac surgery (EVE) clinical trial development: engagement methods using body mapping and virtual consensus building
Source: Front Rehabil Sci. 2026 Oct 8;7:1801084. doi: 10.3389/fresc.2026.1801084 (PMC13686985; doi:10.3389/fresc.2026.1801084)
Supplement: Supplementary file 1 [file Supplementaryfile1.docx]

**Understanding the Lived Experience of Early Mobility Using Body Mapping**

Table of Contents

[Background & Objectives	2](#_Toc201412948)

[People Involved	2](#_Toc201412949)

[Process	3](#_Toc201412950)

[Preparatory Work	3](#_Toc201412951)

[Workshop 1 (3 x 3-hour long sessions)	3](#_Toc201412952)

[***Proposed Agenda: Session 1***	4](#_Toc201412953)

[***Proposed Agenda: Session 2***	6](#_Toc201412954)

[***Proposed Agenda: Session 3***	6](#_Toc201412955)

[Workshop 2 (4 hours)	7](#_Toc201412956)

[Materials Provided to Participants	7](#_Toc201412957)

[***Proposed Agenda***	7](#_Toc201412958)

[Workshop 3 (2.5 hours)	9](#_Toc201412959)

[***Proposed Agenda***	9](#_Toc201412960)

[Debriefing	11](#_Toc201412961)

[Appendix A: Materials Provided Prior to Workshop 1	15](#_Toc201412962)

[Appendix B: Guidelines For Virtual Interactions	17](#_Toc201412963)

# **Background & Objectives**

There is a lack of literature sharing the experience of early mobility of patients and their care partners following cardiac surgery (CS) in the intensive care unit (ICU). This silencing of their experiences limits our understanding and capacity to produce research aligned with their experiences and address their health needs. From a pragmatist worldview, this also means that critical knowledge is missing, as experience is required to know.^1,2^ The lack of definition of early mobility in the CS ICU provides the opportunity to produce a definition co-informed by PWLE, clinicians, researchers, and the literature. Successful early mobilization requires an understanding of barriers and facilitators perceived by PWLE so these can be integrated into the implementation of the intervention and support feasibility, acceptability, and fidelity. Finally, outcome measures used to evaluate early mobility interventions should include outcome and experience measures. The **overall goals of this workshop** are to co-create with PWLE partners: 1) an understanding of the experience of mobility in the CS ICU, 2) a definition of early mobility, 3) identify of barriers and facilitators to early mobility, and 4) select outcome and experience measures.

| **Workshop Number** | **Goal** | **Output** |
| --- | --- | --- |
| 1 | Create and co-analyze body maps | Body maps  Videos of discussions of body maps |
| 2 | Co-create a definition of early mobility  Generate a list of barriers, facilitators, outcome, and experience measures | Definition of early mobility  List of barriers, facilitators, outcome and experience measures |
| 3 | Prioritize barriers, facilitators, outcome, and experience measures | Prioritized barriers, facilitators, outcome, and experience measures |

# **People Involved**

We will form a PWLE engagement panel (PEP)^5^ of 8-12 patients and care partners to align with recommendations for group size to facilitate discussion and participation.^6^ The inclusion of care partners was suggested by the current PWLE study partners, who shared the difficulty of remembering the ICU hospitalization and the key role their care partners play in helping them to piece together memories of the time. Patients and care partners will be recruited from the Patient and Family Advisory Councils at St Boniface Hospital (SBH) and the Winnipeg Regional Health Authority, a growing body of previous cardiac patients and care partners from SBH who have participated in previous PEPs, and a database of past CS patients agreeable to further consultation. Inclusion criteria will be older adults over the age of 65 who have undergone or adults over the age of 18 who have had a loved one undergo elective or semi-urgent outpatient CABG, valve CS, or a combination of the two at SBH in the last five years, are cognitively able to participate in consultation workshop, have access to the internet and an electronic device, are willing to reflect on personal experiences relating to CS and share these in a group setting, can speak and read English, and consent to be recorded.

**Administrative Considerations**

**Team Members & Roles**

Facilitators: Emily, Sheila, April, Kathy

Technology Support: Jacqueline

**Equipment/ Supplies**

- Room with large tables
- Paper 6 feet long x 3 feet wide
- Writing paper
- Pens, Pencils
- Coloured construction paper
- Markers
- Crayons, pencil crayons
- Paint
- Scissors
- Tape
- Paint brushes
- Sticky Notes
- Letter stencils
- Glue
- Magazines
- Audio recorder(s)

# **Process**

Body mapping involves creating a life-size tracing of a body, in the position of the person’s choosing, which is then filled in based on reflections to questions shared in the workshop.^13,15–18^ The body maps can be filled in with paint, markers, crayons, collage, or words in whatever way is meaningful to the PWLE creating the body map.^13,15–18^ This diversity in how reflections are shared supports the shift in power from researchers to PWLE, as PWLE determine how they will share their knowledge.^13,15–18^ The combination of creativity and reflection^15–19^ leads to a deeper, embodied self-awareness and understanding of identity and knowledge created.^16^

## **Preparatory Work**

Prior to the formal workshops, the consent form will be shared, either a physical or electronic copy. The consent form will be signed prior to the commencement of the first session of the first workshop. Demographic information will be collected at this time, including age, self-identified ethnicity, and CS history.^20^ As recommended,^21,22^ participants will be asked about assigned sex at birth and their gender identity. To examine gender as a continuous variable, PWLE will complete the validated GENESIS-PRAXY Gender Questionnaire.^23^

## **Workshop 1 (3 x 3-hour long sessions)**

To reduce the group size and to maximize the time for each PWLE to share their experience, the larger PEP will be split in half and each half will attend one of two body mapping workshops. These workshops will occur over 3 consecutive days. In each workshop, PWLE will be asked to share their experiences of early mobility and reflect on what that experience felt like through the creation of a body map. Following the creation of the body maps, participants will be asked to share an explanation of what they have depicted on their body maps in an video-recorded group discussion.^15–19^ This process will provide an understanding of the experience of PWLE of early mobility and will begin to co-create a definition of early mobility.

**Materials Provided to Participants**

See Appendix A for the documents shared with participants prior to Workshop 1.

### ***Proposed Agenda: Session 1***

a. **Welcome, land acknowledgement, and meeting overview** (5 min activity) (5 mins total) *Led by Emily*

- **Recording reminder:** Remind everyone they’re being recorded
- **Land acknowledgement:** On behalf of all of us here today living on the land of the Indigenous people, we would like to acknowledge that the University of Manitoba campuses are located on the original lands of Anishinaabeg, Cree, Oji-Cree, Dakota, and Dene peoples, and on the homeland of the Métis Nation. We respect the Treaties that were made on these territories, we acknowledge the harms and mistakes of the past, and we dedicate ourselves to move forward in partnership with Indigenous communities in a spirit of reconciliation and collaboration.

b. **Introductions** (5 min activity) (10 mins total) *Led by April & Sheila*

- Initiated by Emily, then everyone introduces themselves moving counter-clockwise
- You were asked to bring a small item that is important to you – can you show us that item and share why you picked it?
- Following introduction share insights on how each person has a different important item and rationale for that favourite which reflects the diversity of our thoughts and experiences. This exercise also started our journey in putting meaning into an item which is embodiment in action and is what we are doing together!

c. **Creating a Safe and Creative Space and housekeeping** (5 min activity) (15 mins total) *Led by Kathy*

- Confidentiality, no “right” answers, listening to others, not speaking over each other
- Location of bathrooms
- Supports available as required

d. **Presentation** (10 min activity) (25 mins total) *Led by Emily*

- Why are we here, what are we doing, what do we hope to achieve, why it’s important
- Talk about CS program and approach – not researching you but engaging you to 1) co-create an understanding of the experience of mobility in the CS ICU, 2) a definition of early mobility, 3) identify of barriers and facilitators to early mobility, and 4) select outcome and experience measures.
- Target endpoints – today and overall
- Joint ownership of study findings:
  - Possibilities include:
    - Authorship through a group name or specific name listed in the acknowledgements
    - After the study, can share name and contact info of participants who are interested in potential future collaborations as well as to further explore the ideas we come up with through this workshop.

e. **Activity #1: Reflections on Early Mobility** (60 min activity) (85 mins total)

- QUESTION: We ask you to think back to your time in ICU, which may be a bit fuzzy and even overwhelming. When you reflect on the experience, we ask you to focus in on your first experiences of mobilization – can you tell us about that experience and what was happening?
  - Prompts (always try to get at the why behind answers):
    - What do you remember thinking?
    - How did you feel physically?
    - How did you feel mentally?
    - How did you feel emotionally?
    - What words would you use to describe mobilizing?
    - What stood out to you?
  - PEP members will reflect on the above questions and begin to determine what they wish to share about their experience. This can occur through writing, drawing, conversation recorded on audio recorder or another way that is meaningful to the PWLE.

f. **BREAK** (10 min activity) (95 mins total)
g. **Activity # 2: Body Mapping Planning** (60 min activity) (155 mins total)

- Question: Next, we want to explore how to move these stories into body maps. To support our work tomorrow, we ask you to first consider the position of your body. What position do you want your arms in? Beside your torso, by your head? What about your legs? How do you want to share your reflections via your body map? What images come to mind when you reflect on your experience?
  - Prompts (always try to get at the why behind answers):
    - What position do you want your body to be in?
    - What colours do you want to use?
    - What textures do you want to use?
    - How will you explain the meaning of what you share via a legend?
    - What supplies do you need that are not available?

h. **BREAK** (10 min activity) (165 mins total)
i. **Group check-in** (10 min activity) (175 mins total) *led by Sheila*

- How is everyone feeling after the reflections?
- Are there any ah-ha moments that you wish to share?
- If you wish, you can draw your initial response to planning body mapping.

j. **Closing remarks and evaluation** (5 min activity) (180 mins total) *led by* *April & Kathy*

- Thank all for their time and reflections
- Share plan for next day

k. Debrief

- Once all PEP members have left, the facilitators will debrief and reflect on the questions posed in Figure 1. These learnings will be applied to future meetings.

### ***Proposed Agenda: Session 2***

a. **Welcome, land acknowledgement, and meeting overview** (10 min activity) (10 mins total) *Led by Emily*

- **Recording reminder:** Remind everyone they’re being recorded
- **Land acknowledgement:** On behalf of all of us here today living on the land of the Indigenous people, we would like to acknowledge that the University of Manitoba campuses are located on the original lands of Anishinaabeg, Cree, Oji-Cree, Dakota, and Dene peoples, and on the homeland of the Métis Nation. We respect the Treaties that were made on these territories, we acknowledge the harms and mistakes of the past, and we dedicate ourselves to move forward in partnership with Indigenous communities in a spirit of reconciliation and collaboration.

b. **Activity: Body Map Creation** (155 min activity) (165 mins total)

- QUESTION: Based on your reflections and planning from yesterday, how do you want your body positioned to show your experience of early mobility in the CS ICU?
  - Facilitators and PEP members to help with tracing the outline of bodies.
  - All art supplies requested will be available.
  - Breaks throughout as required.

c. **Group check-in** (10 min activity) (175 mins total) *led by* *Sheila*

- How is everyone feeling after the reflections?
- Are there any ah-ha moments that you wish to share?
- From yesterday's work and your body mapping creation, is there anything in this process that intrigues you, surprises you, and is concerning to you…Why?

d. **Closing remarks and evaluation** (5 min activity) (180 mins total) *led by* *Kathy & April*

- Thank all for their time and reflections
- Share plan for next day

e. **Debrief**

- Once all PEP members have left, the facilitators will debrief and reflect on the questions posed in Figure 1. These learnings will be applied to future meetings.

### ***Proposed Agenda: Session 3***

a. **Welcome, land acknowledgement, and meeting overview** (5 min activity) (5 mins total) *Led by Emily*

- **Recording reminder:** Remind everyone they’re being video-recorded
- **Land acknowledgement:** On behalf of all of us here today living on the land of the Indigenous people, we would like to acknowledge that the University of Manitoba campuses are located on the original lands of Anishinaabeg, Cree, Oji-Cree, Dakota, and Dene peoples, and on the homeland of the Métis Nation. We respect the Treaties that were made on these territories, we acknowledge the harms and mistakes of the past, and we dedicate ourselves to move forward in partnership with Indigenous communities in a spirit of reconciliation and collaboration.

b. **Activity: Sharing Body Maps** (125 min activity) (130 mins total) (approx.20 min/ PWLE)

- QUESTION: Can you show and explain your body map to us?

c. **BREAK** (10 min activity) (140 mins total)
d. **Group discussion** (30 min activity) (150 mins total) *led by Sheila*

- Bringing it all together**:** what final thoughts and reflections would folks like to share after hearing from the whole group today?

e. **Closing remarks and evaluation** (5 min activity) (180 mins total) *led by April & Kathy*
A. Debrief

Once all panel members have left, the facilitators and the rest of the research team will debrief and reflect on the questions posed in Figure 1. These learnings will be applied to future meetings.

## **Workshop 2 (4 hours)**

Audio-recorded group discussions, including the entire PEP, will continue virtually on MS Teams to refine the co-created definition of early mobility, identify barriers and facilitators to early mobility, and select outcome and experience measures to support intervention evaluation.

## **Materials Provided to Participants**

To facilitate group discussions and share the experiences of early mobility among PWLE who attended each body mapping session, the PEP will be presented with summaries from the body mapping workshop and an overview of outcome and experience measures. These will be generated from the discussions from Workshop 1 Session 3.

### ***Proposed Agenda***

a. **Welcome, land acknowledgement, and meeting overview** (5 min activity) (5 mins total) *Led by Emily*

- **Recording reminder:** Remind everyone they’re being recorded
- **Land acknowledgement:** On behalf of all of us here today living on the land of the Indigenous people, we would like to acknowledge that the University of Manitoba campuses are located on the original lands of Anishinaabeg, Cree, Oji-Cree, Dakota, and Dene peoples, and on the homeland of the Métis Nation. We respect the Treaties that were made on these territories, we acknowledge the harms and mistakes of the past, and we dedicate ourselves to move forward in partnership with Indigenous communities in a spirit of reconciliation and collaboration.

b. **Introductions** (5 min activity) (10 mins total)

- Initiated by Emily, then everyone introduces themselves moving counter-clockwise
- Please tell us your name and a boring detail about yourself.

c. **Ground rules** (5 min activity) (15 mins total) *Led by April?*

- Via Appendix B

d. **Presentation** (10 min activity) (25 mins total) *Led by Emily*

- Why are we here, what are we doing, what do we hope to achieve, why it’s important
- Share overview of body mapping and main takeaways
- Target endpoints – today and overall
- Joint ownership of study findings:
  - Possibilities include:
    - Authorship through a group name or specific name listed in the acknowledgements
    - After the study, can share name and contact info of participants who are interested in potential future collaborations as well as to further explore the ideas we come up with through this workshop

e. **Activity #1: What Early Mobility Means and How It Should Be Defined** (60 min activity) (85 mins total)

- QUESTION: What does “early mobility” mean you?
  - Prompts (always try to get at the why behind answers):
    - When does it start?
    - What does it look like?
    - What does it feel like?
    - How would you explain early mobility to another patient?
  - Suggested approach to ensure that the group doesn’t get stuck on one point: round-robin format – ask everyone to state two to three ideas, next person indicate the ideas that they agreed with (have facilitator put checks beside them), and then add any others that haven’t already been shared. Next round, everyone will have the opportunity to build upon any of the ideas that have been shared. Then will go to open sharing format (box analogy – start with outlining edges, then shading details in).

f. **BREAK** (10 min activity) (95 mins total)
g. **Activity # 2:** **Barriers and Facilitators** (60 min activity) (155 mins total)

- QUESTION: Based on our previous discussion about what early mobility is, what are the barriers to early mobility? What about facilitators?
  - Prompts (always try to get at the why behind answers):
    - Why is this a barrier?
    - Why is this a facilitator?
    - Is the opposite true? Would the opposite of a barrier be a facilitator?
  - Suggested approach to ensure that the group doesn’t get stuck on one point: round-robin format – ask everyone to state two to three ideas, next person indicate the ideas that they agreed with (have facilitator put checks beside them), and then add any others that haven’t already been shared. Next round, everyone will have the opportunity to build upon any of the ideas that have been shared. Then will go to open sharing format (box analogy – start with outlining edges, then shading details in).

h. **BREAK** (10 min activity) (165 mins total)
i. **Activity # 3: Outcome and Experience Measures** (60 min activity) (225 mins total)

- QUESTION: What outcome and experience measures are important when we evaluate early mobility research?
  - Prompts (always try to get at the why behind answers):
    - Does it cover everything you felt/ experienced?
    - How would you explain the importance of that outcome/ experience to a researcher?
    - What does that outcome mean to you?
    - What does that experience measure mean to you?
  - Suggested approach to ensure that the group doesn’t get stuck on one point: round-robin format – ask everyone to state two to three ideas, next person indicate the ideas that they agreed with (have facilitator put checks beside them), and then add any others that haven’t already been shared. Next round, everyone will have the opportunity to build upon any of the ideas that have been shared. Then will go to open sharing format (box analogy – start with outlining edges, then shading details in).

j. **Closing remarks and evaluation** (5 min activity) (230 mins total) *Facilitated by*
k. **Debrief**

Once all panel members have left, the facilitators and rest of the research team will debrief and reflect on the questions posed in Figure 1. These learnings will be applied to future meetings.

## **Workshop 3 (2.5 hours)**

Following these discussions, we will begin the refinement towards prioritizing barriers and facilitators along with selecting outcome and experience measures. Consensus techniques will be used to prioritize the barriers, facilitators, outcome and experience measures. Similar to a project completed by members of my committee,^24^ a thematic summary of the PEP-identified barriers, facilitators, outcome and experience measures will be presented and a modified Delphi technique^25,26^ will be used to prioritize the barriers and facilitators and then the outcome and experience measures. This virtual session will use an online survey to allow for the anonymity of responses.^25,26^ Two rounds of prioritization will occur via Google Forms with a discussion between focused on the initial prioritization from least agreement to most agreement.^24^

**Materials Provided to Participants**

The research team will generate a thematic summary of barriers, facilitators, and outcome and experience measures based on the transcript from the group discussion.

### ***Proposed Agenda***

a. **Welcome, land acknowledgement, and meeting overview** (5 min activity) (5 mins total) *Led by Emily*

- **Recording reminder:** Remind everyone they’re being recorded
- **Land acknowledgement:** On behalf of all of us here today living on the land of the Indigenous people, we would like to acknowledge that the University of Manitoba campuses are located on the original lands of Anishinaabeg, Cree, Oji-Cree, Dakota, and Dene peoples, and on the homeland of the Métis Nation. We respect the Treaties that were made on these territories, we acknowledge the harms and mistakes of the past, and we dedicate ourselves to move forward in partnership with Indigenous communities in a spirit of reconciliation and collaboration.

b. **Ground rules** (5 min activity) (10 mins total) *Led by Kathy*
c. **Activity #1: Prioritization of Barriers and Facilitators** (20 min activity) (35 mins total)

- REQUEST: Using the Google Forms link emailed to you, please prioritize the barriers and then the facilitators from most to least important.

d. **Brief analysis of responses** (10 min activity) (45 min total) by the research team
e. **Group discussion** (20 min activity) (65 mins total) *led by Emily*

- Beginning with the least agreed-upon barriers, why are these not important? Why are others more important?
- Beginning with the least agreed-upon facilitators, why are these not important? Why are others more important?

f. **Activity #2: Re-Prioritization of Barriers and Facilitators** (20 min activity) (85 mins total)

- REQUEST: Using the Google Forms link emailed to you, please prioritize the barriers and then the facilitators from most to least important.

g. **BREAK** (10 min activity) (95 mins total)
h. **Activity #3: Prioritization of Outcome and Experience Measures** (20 min activity) (105 mins total)

- REQUEST: Using the Google Forms link emailed to you, please prioritize the outcome and experience measures from most to least important.

i. **Brief analysis of responses** (10 min activity) (115 min total) by the research team
j. **Group discussion** (20 min activity) (135 mins total) *led by Emily*

- Beginning with the least agreed-upon outcome measures, why are these not important? Why are others more important?
- Beginning with the least agreed-upon experience measures, why are these not important? Why are others more important?

k. **Activity #3: Prioritization of Outcome and Experience Measures** (20 min activity) (155 mins total)

- REQUEST: Using the Google Forms link emailed to you, please prioritize the outcome and experience measures from most to least important.

l. **Closing remarks and evaluation** (5 min activity) (160 mins total) *led by April*
m. **Debrief**

Once all panel members have left, the facilitators and rest of the research team will debrief and reflect on the questions posed in Figure 1. These learnings will be applied to future meetings.

## **Debriefing**

What was supposed to happen?

What actually happened?

Why were there differences?

Who does what as a result?

What can we learn?

Figure 1. After action review. Modified from: <https://workshopbank.com/after-action-review-aar>

References

1.	Allemang B, Sitter K, Dimitropoulos G. Pragmatism as a paradigm for patient‐oriented research. *Health Expectations*. 2022;25(1):38-47. doi:10.1111/hex.13384

2.	Biesta G. Pragmatism and the Philosophical Foundations of Mixed Methods Research. In: *SAGE Handbook of Mixed Methods in Social & Behavioral Research*. ; 2015:95-118. doi:10.4135/9781506335193.n4

3.	Staniszewska S, Brett J, Simera I, et al. GRIPP2 reporting checklists: tools to improve reporting of patient and public involvement in research. *BMJ*. Published online August 2, 2017:j3453. doi:10.1136/bmj.j3453

4.	Heidari S, Babor TF, De Castro P, Tort S, Curno M. Sex and Gender Equity in Research: rationale for the SAGER guidelines and recommended use. *Res Integr Peer Rev*. 2016;1(1):2. doi:10.1186/s41073-016-0007-6

5.	Arkind J, Likumahuwa-Ackman S, Warren N, et al. Lessons learned from developing a patient engagement panel: An OCHIN report. *Journal of the American Board of Family Medicine*. 2015;28(5):632-638. doi:10.3122/jabfm.2015.05.150009

6.	Patient-Centered Outcomes Research Institute. Patient advisory panels FAQs. Published 2020. https://www.pcori.org/engagement/engage-us/pcoris-advisory-panels/pcori-advisory-panels-faqs

7.	Nguyen Q, Uminski K, Hiebert BM, Tangri N, Arora RC. Midterm outcomes after postoperative delirium on cognition and mood in patients after cardiac surgery. *Journal of Thoracic and Cardiovascular Surgery*. 2018;155(2):660-667.e2. doi:10.1016/j.jtcvs.2017.09.131

8.	International Association for Public Participation. IAP2 Public Participation Spectrum. *International Association for Public Participation*. Published online 2018. https://cdn.ymaws.com/www.iap2.org/resource/resmgr/foundations_course/IAP2_P2_Spectrum_FINAL.pdf

9.	George & Fay Yee Centre for Healthcare Innovation. Methods of Patient & Public Engagement: A guide. *Strategy for Patient Oriented Research*. Published online 2020:1-31.

10.	Canadian Institutes of Health Research. *Strategy for Patient-Oriented Research: Patient Engagement Framework*.; 2014:1-11. http://www.cihr-irsc.gc.ca/e/48413.html, Accessed on April 26, 2015.

11.	Chudyk AM, Horrill T, Waldman C, et al. Scoping review of models and frameworks of patient engagement in health services research. *BMJ Open*. 2022;12(8):e063507. doi:10.1136/bmjopen-2022-063507

12.	Oravec N, Arora RC, Bjorklund B, et al. Patient and caregiver preferences and prioritized outcomes for cardiac surgery: A scoping review and consultation workshop. *The Journal of Thoracic and Cardiovascular Surgery*. Published online November 2021:1-12. doi:10.1016/j.jtcvs.2021.11.052

13.	Boydell KM, ed. *Applying Body Mapping in Research: An Arts-Based Method*. Routledge; 2021.

14.	Leavy P. *Method Meets Art*. 3rd ed. Guilford Press; 2020.

15.	Macken S, Nathan S, Jersky M, Boydell KM, Gibson A. Body Mapping in a Drug and Alcohol Treatment Program: Eliciting New Identity and Experience. *International journal of environmental research and public health*. 2021;18(9). doi:10.3390/ijerph18094942

16.	Boydell KM, Bennett J, Dew A, et al. Women and stigma: A protocol for understanding intersections of experience through body mapping. *International Journal of Environmental Research and Public Health*. 2020;17(15):1-10. doi:10.3390/ijerph17155432

17.	Brodyn A, Lee SY, Futrell E, et al. Body Mapping and Story Circles in Sexual Health Research With Youth of Color: Methodological Insights and Study Findings From Adolescent X, an Art-Based Research Project. *Health Promotion Practice*. Published online 2021:1-15. doi:10.1177/15248399211039796

18.	Ryan S, Ussher JM, Hawkey A. Managing the premenstrual body: a body mapping study of women’s negotiation of premenstrual food cravings and exercise. *Journal of Eating Disorders*. 2021;9(1):1-14. doi:10.1186/s40337-021-00478-6

19.	de Souza J, Ventura CAA, de Oliveira JLG, et al. Experience of vulnerable women narrated through the body-mapping technique. *International Journal of Environmental Research and Public Health*. 2021;18(24). doi:10.3390/ijerph182413094

20.	Chudyk AM, Ragheb S, Kent D, et al. Patient Engagement in the Design of a Mobile Health App That Supports Enhanced Recovery Protocols for Cardiac Surgery: Development Study. *JMIR Perioper Med*. 2021;4(2):e26597. doi:10.2196/26597

21.	Park A. Reachable: Data collection methods for sexual orientation and gender identity.

22.	Canadian Institutes of Health Research Institute of Gender and Health. *Science Is Better with Sex and Gender Strategic Plan 2018-2023*.; 2018:1-40.

23.	Pilote L. GENESIS PRAXY Gender Questionnaire. Published online 2015.

24.	Oravec N, Schultz ASH, Bjorklund B, et al. A Virtual , Multi-Session Workshop Model for Integrating Patient and Public Perspectives in Research Analysis and Interpretation. *International Journal of Qualitative Methods*. 2022;21:1-12. doi:10.1177/16094069221124402

25.	Normand SLT, McNeil BJ, Peterson LE, Heather Palmer R. Eliciting expert opinion using the Delphi technique: Identifying performance indicators for cardiovascular disease. *International Journal for Quality in Health Care*. 1998;10(3):247-260. doi:10.1093/intqhc/10.3.247

26.	Powell C. The Delphi technique: Myths and realities. *Journal of Advanced Nursing*. 2003;41(4):376-382. doi:10.1046/j.1365-2648.2003.02537.x

27.	Rose G. “The Good Eye”: Looking at Pictures Using Compositional Interpretation. In: Rojek R, ed. *Visual Methodologies: An Introduction to Researching with Visual Materials*. 4th ed. SAGE Publications Ltd; 2016:56-84.

28.	Kim JH. *Understanding Narrative Inquiry: The Crafting and Analysis of Stories as Research*. (Salmon H, ed.). SAGE Publications Ltd; 2016.

29.	McMaster University. Public and Patient Engagement Evaluation Tool (PPEET) version 2.0. Published online 2018. Accessed April 29, 2023. https://ppe.mcmaster.ca/wp-content/uploads/2023/04/PPEET_Version-2.0_Full-Set.pdf

30.	Ekegren CL, Mather AM, Reeder S, Kimmel L, Ashe MC, Gabbe BJ. Can a new ward environment and intensive allied health staffing model enhance therapeutic opportunities in trauma care? A behavioural mapping study of patients’ activities and interactions. *Clin Rehabil*. 2022;36(10):1314-1323. doi:10.1177/02692155221107739

31.	Slade SC, Dionne CE, Underwood M, et al. Consensus on Exercise Reporting Template (CERT): Modified Delphi Study. *Physical Therapy*. 2016;96(10):1514-1524. doi:10.2522/ptj.20150668

32.	Polit DF, Beck CT. *Nursing Research: Generating and Assessing Evidence for Nursing Practice.* 10th ed. Wolters Kluwer; 2017.

# **Appendix A: Materials Provided Prior to Workshop 1**

**In-person workshops**

**WHERE**:

**IH Asper Institute (see attached map)**

Please keep your receipts. We will reimburse you for your parking.

**WHAT TO WEAR:**

Wear comfortable clothes and shoes- or even bring slippers. This is important as you will be moving around, laying down, possibly bending.

**WHEN:**

**Workshop 1: You will meet everyone, and we will discuss body mapping.**

**June 4th-** 9am to 12pm

We will be doing an icebreaker exercise together to get to know one another and to start thinking artistically. Please bring a small item (something you can hold in your hand) that is important to you. We will ask everyone to do this and share why it is important in the workshop. This does not have to relate to your surgery, it can be anything you feel is important.

**You will be split into two groups for the next two sessions to ensure everyone has time to speak and share.**

**Workshop 2: You will create your body map.**

**June 5th-** 9 am to 12 pm

**Workshop 3: You will share your body map.**

**June 6th-** 9 am to 12 pm OR

**Virtual workshops**

**WHERE**:

**Online-** Microsoft Teams- We will provide you with assistance with how to log on.

**Workshop 4:** **You will discuss what early mobility means to you, the barriers and facilitators to it, and how it should be defined and measured.**

**June 14th-** 8:30 am to 12:30 pm

**Workshop 5: You will participate in a prioritization activity. This is a process used to rank or order items based on their level of importance to help us decide where to focus their time, resources, and efforts to improve early mobility in cardiac surgery.**

**June 25th-** 9 am to 11:30 am

**Body Mapping Sample Questions**

We are going to share the questions that will guide the creation of your body map. We are doing this not because we want you to do work before the workshop, but because we want to give you time to think about your experience, what you want to share, and how you want to share it. We will talk about these in the first workshop as well.

**QUESTION:** We ask you to think back to your time in ICU, which may be a bit fuzzy and even overwhelming. When you reflect on the experience, we ask you to focus in on your first experiences of mobilization – can you tell us about that experience and what was happening?

- Prompts (always try to get at the why behind answers):
  - What do you remember thinking?
  - How did you feel physically?
  - How did you feel mentally?
  - How did you feel emotionally?
  - What words would you use to describe mobilizing?
  - What stood out to you?
- Begin to determine what you wish to share about your experience. This can occur through writing, drawing, conversation recorded on audio recorder or another way that is meaningful to YOU.

Next, we want to explore how to move these stories into body maps. To support our work tomorrow, we ask you to first consider the position of your body. What position do you want your arms in? Beside your torso, by your head? What about your legs? How do you want to share your reflections via your body map? What images come to mind when you reflect on your experience?

- Prompts (always try to get at the why behind answers):
  - What position do you want your body to be in?
  - What colours do you want to use?
  - What textures do you want to use?
  - How will you explain the meaning of what you share via a legend?
  - What supplies do you need that are not available?

# **Appendix B: Guidelines For Virtual Interactions**

During our meetings, please:

1) Arrive at least five minutes early so that we can start and end on time.
2) Keep your audio muted until you are ready to speak.
3) In order to ensure that everyone has room to express themselves, please raise a “virtual” hand or wave your actual hand (if your camera is on) to get a facilitator’s attention if you’d like to say something.
4) Speak up! We want to hear from everyone.
5) Share the airtime. We want to ensure that everyone has the opportunity to speak.
6) Be respectful of others’ viewpoints.

- If someone says something that you disagree with, instead of telling them that they’re wrong, ask the questions of why they think it works (e.g., “Help me understand – how would that work or how has that worked for you?”).

7) Actively participate.
8) Keep to the topic under discussion as we have limited time together.
9) Respect confidentiality where appropriate.
10) Let us know when you do not want to answer a question or share an experience.
11) Avoid use of chat function
12) Did we miss anything???
